# Impact of Sliding Window Length in Indoor Human Motion Modes and Pose Pattern Recognition Based on Smartphone Sensors

**DOI:** 10.3390/s18061965

**Published:** 2018-06-18

**Authors:** Gaojing Wang, Qingquan Li, Lei Wang, Wei Wang, Mengqi Wu, Tao Liu

**Affiliations:** 1 State Key Laboratory of Information Engineering in Surveying, Mapping and Remote Sensing, Wuhan University, Wuhan 430079, China; wanggaojing@whu.edu.cn; 2 School of Geodesy and Geomatics, Wuhan University, Wuhan 430079, China; wanngwei@whu.edu.cn (W.W.); mengqi.wu@whu.edu.cn (M.W.); 3 College of Resources and Environment, Henan University of Economics and Law, Zhengzhou 450002, China; liuzimo@whu.edu.cn

**Keywords:** human motion mode, human pose pattern, window length, machine-learning method, smartphone sensors

## Abstract

Human activity recognition (HAR) is essential for understanding people’s habits and behaviors, providing an important data source for precise marketing and research in psychology and sociology. Different approaches have been proposed and applied to HAR. Data segmentation using a sliding window is a basic step during the HAR procedure, wherein the window length directly affects recognition performance. However, the window length is generally randomly selected without systematic study. In this study, we examined the impact of window length on smartphone sensor-based human motion and pose pattern recognition. With data collected from smartphone sensors, we tested a range of window lengths on five popular machine-learning methods: decision tree, support vector machine, K-nearest neighbor, Gaussian naïve Bayesian, and adaptive boosting. From the results, we provide recommendations for choosing the appropriate window length. Results corroborate that the influence of window length on the recognition of motion modes is significant but largely limited to pose pattern recognition. For motion mode recognition, a window length between 2.5–3.5 s can provide an optimal tradeoff between recognition performance and speed. Adaptive boosting outperformed the other methods. For pose pattern recognition, 0.5 s was enough to obtain a satisfactory result. In addition, all of the tested methods performed well.

## 1. Introduction

Human activity recognition (HAR) has become a popular research topic. Analyzing human activities is an effective method for understanding the human context, living habits, and demands [1,2,3,4,5,6,7,8,9,10,11,12,13,14]. HAR can be used in many applications, such as precise marketing and human psychology. Scholars regard human motion, such as walking, being at rest, and riding an elevator, and posing, which includes activities such as calling and typing, as two highly interesting types of human activity [15,16]. These activities are particularly important for pedestrian navigation applications [17], because they support the robustness and accuracy of the navigation. Varying motion modes and pose patterns require different algorithms and constraints to obtain accurate positioning results [18]. For instance, when walking is detected, users’ vertical locations should be fixed, whereas horizontal displacement and direction must be updated. When riding an elevator is detected, the horizontal location should be fixed, whereas the vertical location must be updated. When using an escalator is detected, horizontal and vertical displacements should be updated. Moreover, the models of misalignment estimation (i.e., differentiating between pedestrian heading and smartphone orientation) differ for each motion and pose [19]. Therefore, awareness of user motion modes and pose patterns can determine the correct misalignment estimation model, and potentially improve positioning solutions. Additionally, the optimal type of sensor during positioning varies for each human pose [20]. For instance, a gyroscope is an optimal sensor for pedestrian dead reckoning (PDR) when users carry their smartphone in a trouser pocket. However, an accelerometer is an excellent option for phoning and typing recognition. HAR also provides guidance measures for patient treatment, and has thus attracted increased attention in the medical treatment field [21].

Considerable research has been conducted on human motion modes and pose pattern recognition. Researchers initially focused on the combinations of motion modes for motion mode recognition. Yang et al. [22] considered four motion modes: sitting, standing, walking, and running. Prasertsung et al. [23] focused on rising and falling modes, involving stairs. Choudhary et al. [24] proposed the vertical motion mode by presenting an elevator case; Bao et al. [25] focused on the complicated motion mode of riding escalators; and Elhoushi et al. [17] introduced the detection of walking transitioning to the escalator motion mode. However, much of the early research on motion modes focused on extracting motion information from wearable motion sensors that were attached to certain body parts [26,27,28,29,30,31]. This research field has leveraged the emergence of smartphones, because such devices are equipped with powerful micro-processing units and high-quality, versatile sensors. The smartphone is more acceptable for users than wearable sensors, because smartphones can operate as a multi-purpose personal assistant, whereas wearable sensors only meet specific demands [32,33]. Therefore, this study focused on smartphone sensor-based motion modes and pose pattern recognition.

Machine-learning methods are typically adopted as classifiers in the research on human motion and pose recognition [21,22,23]. When used to detect human motion modes and pose patterns, all sensor data should first be segmented using a windowing method and classified at every segmentation. Therefore, selecting the window length directly affects classification performance. A small window accelerates recognition but may negatively impact recognition performance. As such, the tradeoff between recognition performance and latency must be carefully considered during the algorithm design. User requirements should also be considered during this phase. Several applications aimed for excellent classification performance regardless of speed (e.g., counting the number of steps in a day [34,35]), whereas others aimed to create speed-critical applications (e.g., real-time positioning). Therefore, understanding the effect of window length on human motion modes and pose pattern recognition can help select the suitable window length during the algorithm design to meet specific user requirements.

In previous research, several techniques for data segmentation that divide sensor signals into usable small parts have been developed [36,37,38,39,40,41]. Among the known techniques, the sliding window approach is the most widely employed [42,43,44,45], being regarded as the best approach for research given its simplicity and stability, and a wide range of window lengths has been used in past studies. Windows as short as 0.5 s and 0.8 s were used to recognize walking, jogging, and going up or down the stairs [46], whereas a window of 1 s with a decision tree (DT) [47] was used to classify stationary, walking, running, and biking motion modes. Additionally, with a neural network [48], a window of 2 s was adopted to classify the motion modes of walking, upstairs and downstairs movement, running, and sitting with varying poses. An average accuracy of 93% was achieved. A window of 5 s was used to classify walking, standing, and climbing stairs using handheld smartphones, where multiple methods achieved a high accuracy score of 84% [49]. A window of 7.5 s was adopted in recognizing walking, stationary, running, and cycling for cases where the smartphone is inside the trouser pocket of users [50], and a classification accuracy score of 93.9% was achieved based on the K-nearest neighbor (KNN) machine learning.

In this paper, we initially examined the influence of window length on human motion modes and pose pattern recognition using five popular machine-learning algorithms. Subsequently, we examined the performance of human motion modes and pose pattern recognition based on different window lengths and machine-learning algorithms using smartphone sensor data. Lastly, the suitable window length for human motion modes and pose pattern recognition is recommended.

The rest of this paper is constructed as follows. Section 2 outlines the methods used for activity classification methodologies. Section 3 describes the experimental setup. Section 4 analyzes the results, which are discussed in Section 5. Section 6 presents the limitations of the study and concludes this paper.

## 2. HAR Workflow

**Figure 1:**
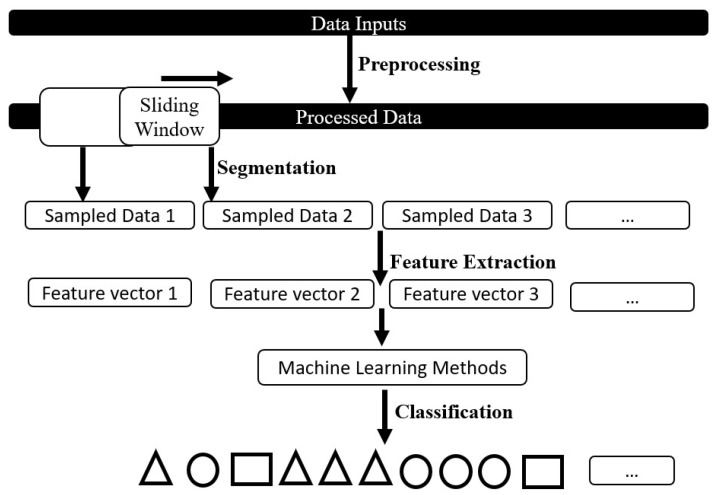
Overview of human activity recognition (HAR) workflow.

Generally, HAR includes four steps: data preprocessing, segmentation, feature extraction, and classification (Figure 1). Sensors can provide multiple data streams for use as data input, such as raw acceleration and air pressure.

The subsequent feature extraction process determines useful features and distinguishes the activities. Feature extraction requires data segments for use as the input data. Thus, raw data streams should be cut into segments. The sliding window segmentation algorithm has been widely used to split sensor data and maximize data usage. Feature extraction is then performed on the data segments. Time-domain statistical and frequency-domain features [23,51,52] are conventionally used as feature input.

The key step in HAR is classification, which takes advantage of the extracted features. Machine-learning methods that can explore unique patterns for classification are popularly used in motion modes and pose pattern recognition. In this study, five machine-learning methods are examined: support vector machine (SVM), KNN, decision tree (DT), Gaussian naïve Bayesian (GNB), and adaptive boosting (Adaboost). The machine-learning methods used in this study are briefly introduced as follows.

The SVM theory was proposed by Vapnik and Chervonenkis [53]. The effectiveness of SVM has been proven to be effective at addressing many problems, such as handwritten digit recognition, face detection in images, and text categorization. SVM achieves high classification accuracy and is robust to noisy data and overfitting problems. Therefore, SVM is considered one of the top classifiers in terms of generalization, and is a popular machine-learning approach in HAR [54,55].

KNN groups feature vectors into clusters that represent different classes [56]. For KNN, the parameter k can be used to regulate underfitting and overfitting. Reducing the value of k increases the sensitivity of the classifier to training data noise, but makes the classifier prone to overfit. Susi et al. [57] achieved accuracy rates ranging from 80% to 84% for upstairs and downstairs movement with k = 1. KNN has also been used in other studies [58,59].

A DT solves a classification problem through a series of cascading decision questions. A feature vector, which satisfies a specific set of questions, is assigned to a specific class. This method is represented graphically using a tree structure, where each internal node is a test on a feature compared with the threshold, and the remaining values refer to the decided classes. Its implementation is based on a loop of if/else conditions. Many types of DTs are generated by different algorithms. In our research, C4.5 was adopted. DTs have been used widely by researchers, many of whom agree that it provides highly accurate results [57].

The naïve Bayesian classifier determines the probability of an event that belongs to a certain class based on Bayesian theorem through using a naïve method [60], assuming that all of the input features are independent. When dealing with continuous data, a typical assumption is that the continuous values associated with each class are distributed according to Gaussian distribution. In such cases, naïve Bayesian (NB) is also called GNB. Compared with other algorithms, the NB classifier is profoundly easy to implement for training and evaluation algorithms. However, this simplicity leads to a much lower accuracy than that of many other classifiers. NB has obtained accuracy rates ranging from 68% to 72% for upstairs and downstairs motion modes, respectively, and 89% to 93% for walking and running, respectively [57].

The adaptive boosting (Adaboost) is a machine-learning meta-algorithm formulated by Yoav Freund and Robert Schapire [61]. It is a method that can be used with other machine-learning methods to improve recognition accuracy. Adaboost combines the outputs of plenty of “weak” classifiers into a weighted sum that represents the final output. AdaBoost is adaptive in the sense that subsequent weak learners are tweaked in favor of those instances misclassified by previous classifiers. The individual learners can be weak, but as long as the performance of each one is slightly better than random guessing, the final model can be proven to converge to a strong learner. It has been proved effective in HAR in previous researches [62,63]. In this paper, Adaboot emended with decision tree (C4.5) was adopted.

In our research, these methods were used to generate robust recognition results. In the training phase, training data streams were segmented with a fixed-length sliding window. Subsequently, features were extracted from data segmentations with equal lengths and fed into the classifiers. When the classifiers are trained and deployed for activity recognition, sensor data streams that must be identified should be sectioned into data segments with the same length as those used for the training data to ensure the effectiveness of the trained classifiers. Understanding the impact of window length on activity recognition can help determine the appropriate window length and present the classification result without bias.

## 3. Experiment Setup

An extensive experiment was performed in our research. The experiment is described in this section.

### 3.1. Data Aquicisiton

We collected a dataset with smartphones in a typical shopping center with nine floors equipped with escalators and elevators. We recruited 10 subjects for the data collection, whose heights ranged from 163 cm to 180 cm and weights ranged from 50 kg to 80 kg. Among the 10 subjects, seven were male and three (subjects 1–3) were female, aged 20 to 30 years old. To protect the privacy and personal information of the subjects, we only show the approximate range of their height and weight in Table 1.

**Figure 2:**
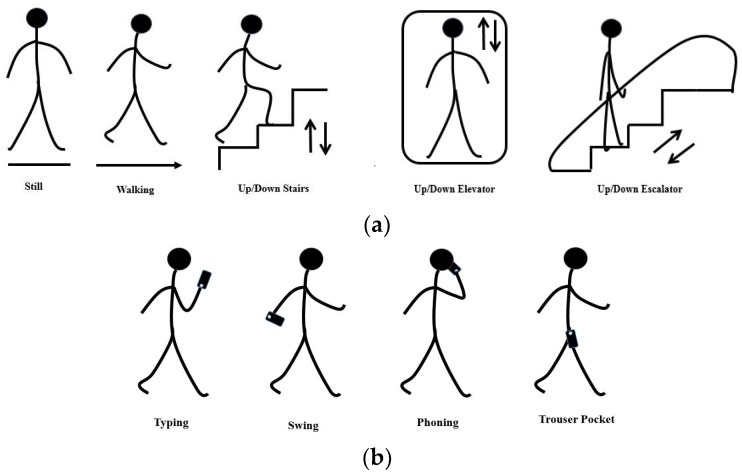
(**a**) Human motion modes and (**b**) pose patterns covered in this study.

**Figure 3:**
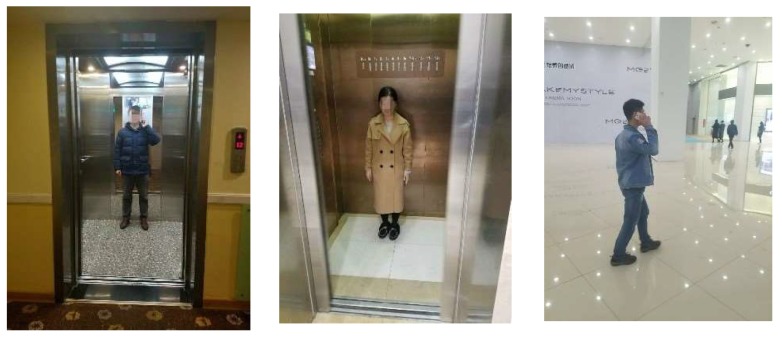

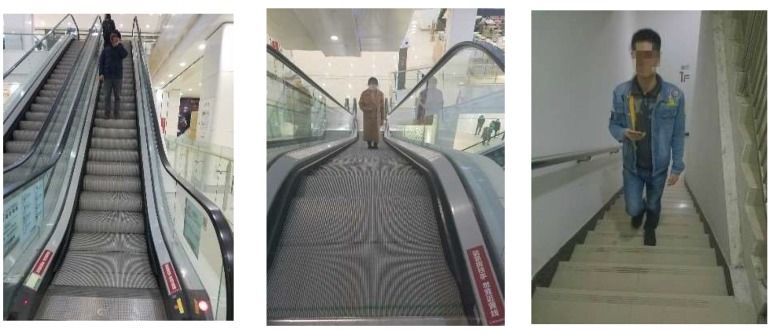
Typical data collection scene. **Upper row**: elevators 1 and 2 and walking. **Lower row**: escalators 1 and 2 and going upstairs (from **left** to **right**).

In daily life, people may use their smartphone in different poses. To increase the robustness of our estimation, and in contrast to previous research, in which smartphones were fixed in one pose [23,51], we considered smartphone usage poses and motion modes when we collected the sensor data. Through observation, we considered eight common indoor pedestrian motion modes and four smartphone usage poses, as shown in Figure 2a,b. Based on these activities and previous works [18,64,65,66], accelerometer, barometer, and gravity sensors equipped in the smartphone were chosen in our research. The sensor data of every motion mode under each pose, and every pose under each motion mode, were collected. Notably, the sensor data of the dynamic motion modes (walking and going up and down stairs) were collected with three walking speeds: slow, normal, and fast. In addition, left-hand and right-hand usage was considered under each pose. Specifically, the left and right trouser pockets were considered in such poses. The data collection campaign lasted over one week, and 21 h of valid test data were collected. Figure 3 demonstrates the data collection scenarios. To protect the privacy, faces of the subjects in Figure 3 were covered with mosaics.

In our experimental settings, walking distance was approximately 500 m, the escalator and stairs descended and ascended between the first and 10th floors, and the elevator covered from the first to the 26th floor. The subjects were blinded to the purpose of the experiment during data collection, and were thus allowed unrestricted smartphone usage to guarantee a natural performance [15,17,67,68,69].

### 3.2. Adopted Sensors and Features

We used acceleration magnitude instead of vector to avoid the negative influence of smartphone orientation on motion mode recognition [23,70]. For pose pattern recognition, gravity sensor data were used to reduce the influence of various motion modes [71]. Actually, the gravity sensor is not a real sensor, but it is obtained by processing data provided by the accelerometer and gyroscope [72]. Hence, motion modes and pose pattern recognition were performed separately and simultaneously.

Table 2 presents a detailed description of the smartphone sensors and measurement types used in this study. In our experiment, we directly used the raw data stream collected at 50 Hz without any specific preprocessing to avoid relevant information loss. During segmentation, a window length ranging from 0.5 s to 7 s with an interval of 0.5 s was adopted for analysis, and the sliding overlap of 0.5 s was used for window sizes larger than 0.5 s. This range largely covers the window lengths used in previous research.

In this study, time-domain and frequency-domain features, which were normally adopted in previous studies [23,51,52], were used for classification (Table 3). These features were extracted for each segment after windowing. Notably, human actions belong to the low-frequency domain, and fast Fourier transformation (FFT) calculations are time-consuming. Thus, we adopted the second to ninth FFT coefficients as the frequency-domain features [52]. We forewent the first FFT coefficient because it represents a direct component, which is similar to the mean value of the sequence. Features were then extracted for every data stream for pattern recognition, as shown in Table 3.

### 3.3. Performance Metric

The F1 score was used as a performance metric to gauge classification performance. It is a combination of precision and recall measures that can represent the detection result with less bias than the accuracy in multi-class classification problems, especially with disproportionate samples in each class [67]. Suppose that in classifying classes A and B, we obtained a confusion matrix (Table 4).

In the matrix, true-positive (TP) is the number of observations that are positive and were predicted to be positive, false-negative (FN) is the number of observations that are positive but were predicted to be negative, true-negative (TN) is the number of observations that are negative and were predicted to be negative, and false-positive (FP) is the number of observations that are negative but were predicted to be positive. Precision, recall, and F1 score are defined as follows:

(1) $$\begin{array}{l} precision=\frac{TP}{TP+FP}=\frac{positive\;predicted\;corretly}{all\;positive\;predictions}\\ recall=\frac{TP}{TP+FN}=\frac{positive\;predicted\;corretly}{all\;positive\;observations}\\ F1=2\cdot \frac{precision\times recall}{precision+recall} \end{array}$$

A high F1 score indicates a high level of classification performance and agreement between the classification and true value.

### 3.4. Validation and Testing Strategy

Ten-fold cross-validation, leave-one-subject-out cross-validation (LOOCV), and boot-strapping strategies have been used in the literature. As previously summarized [73,74,75], LOOCV and bootstrapping are better for risk estimation, whereas 10-fold CV is the most accurate approach for model selection. Chen et al. [52] reported that, in contrast to rest-to-one mode in LOOCV, the all-to-one model better enhances robustness and is recommended for HAR. Therefore, in our study, 10-fold cross-validation was first used to select the machine-learning method parameters [73]. Parameters with higher average F1 scores in the cross-validation were selected. As for SVM, the linear kernel function and radial basis kernel function (RBF) were adopted in parameter selection for their popularity in HAR [53,52]. The parameter searching of k in KNN was performed in a wide range from 1 to 10. The searching range of the number of embedding decision trees in Adaboost was set from 10 to 100.

Finally, the linear kernel and a parameter of two were selected for SVM and KNN, respectively, and 20 decision trees were incorporated in Adaboost. Subsequently, a 100-time bootstrapping strategy was adopted to ensure statistical robustness and produce an asymptotic convergence to the correct estimation of system performance [73].

**Figure 4:**
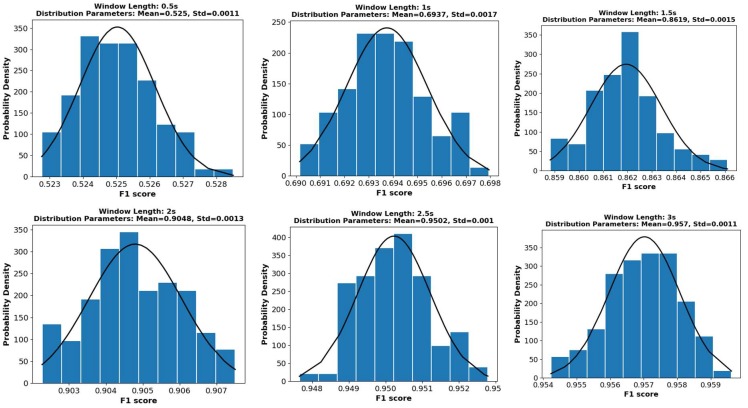
The 100-time bootstrapping results of motion mode recognition using support vector machine (SVM) and different window lengths (0.5–3 s).

We list some of the bootstrapping distribution results in Figure 4 for brevity. These results were derived from the SVM motion mode classification on window sizes ranging from 0.5 s to 3 s. The figure shows the normal distribution fitting curves (black) based on the mean and SD of the bootstrapping results. The results are consistent with normal distribution, and the SD of the 100-time bootstrapping results was less than 1%. In our research, the maximum SD in motion modes and pose pattern recognition was 0.59%. Such a small variation denotes the reliability of our results. The mean values of 100-time bootstrapping results were used as the final results. Section 4 presents the results of motion mode and pose patter classification.

## 4. Experiment Result and Analysis

### 4.1. Motion Mode Classification Result

**Figure 5:**
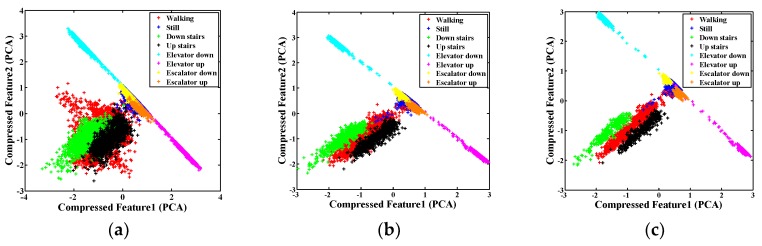
Distribution of compressed features of human motion modes with various window lengths: (**a**) 1 s; (**b**) 2 s; and (**c**) 3 s.

First, we examined the influence of window length on the feature extraction of motion mode recognition. We visualized the compressed features extracted at different window lengths on a two-dimensional plane based on principal component analysis [76]. Figure 5 presents the results. The figure shows that data segments with long window lengths indicate feature separability. Points with the same color are increasingly concentrated with the increase in window length, and the boundaries between the features become evident. This notion is particularly true for walking (red) and going up and down stairs (black and green). When the window length is only one second, these point groups were lumped together. When the window length increased to three seconds, the boundaries between cases emerged. These results reveal the significant effect of window length on the classification of human motion modes. In addition, the linear boundaries among various point groups also prove the good performance of SVM with linear kernel.

#### 4.1.1. Global Evaluation

**Figure 6:**
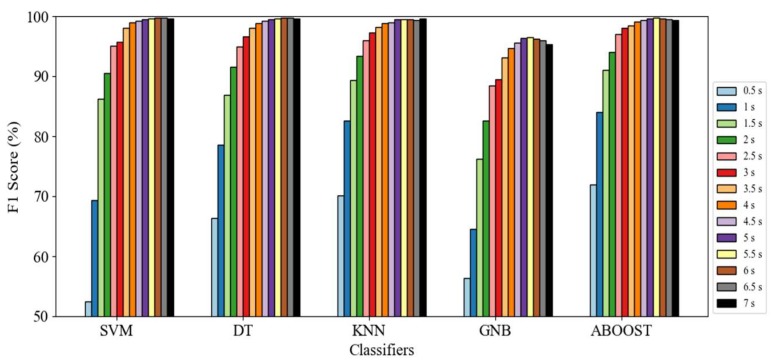
Average F1 score of motion mode classification using different machine-learning methods and window lengths.

We then evaluated the influence of window length on motion mode recognition using the classification results. Figure 6 presents the results. The figure shows the average F1 score of the eight motion modes with window lengths varying from 0.5 s to 7 s using the five classification methods. Initially, we observed that the F1 score considerably increased with the expansion in window length. The SVM F1 score increased significantly from 52.5% to 98% when the window length increased from 0.5 s to 3.5 s. The F1 score improvement using other classification methods was also significant at 66.37% to 98% for DT, 70.14% to 98.12% for KNN, 56.34% to 93.18% for GNB, and 73% to 98.49% for Adaboost (“ABOOST”). These results prove that a notable improvement in classification performance using an increased window length occurs across all of the adopted methods.

Despite the evident benefit of expanding the window length, blindly increasing the window size to improve performance is unreasonable, because the additional benefit of expanding the window length is evidently reduced with the increased in window size. For SVM, the F1 scores increased by less than 1.5% after 3.5 s. To a lesser extent, this result applies to the DT, KNN, GNB, and Adaboost models. In addition, F1 scores decreased when the window length exceeds six seconds.

In a real application, a large window length leads to large recognition latency. However, when we moderately reduced our performance requirement, recognition latency evidently decreased with a minimal tradeoff with recognition performance. If we require an F1 score above 99%, then a sliding window larger than four seconds with SVM, KNN, DT, or Adaboost is satisfactory. However, if the required F1 score decreased to 95%, the window length can be reduced to 2.5 s. If we further lower the required F1 score to 90%, then a window length of two seconds is satisfactory.

In summary, the motion mode classification results show that the impact of the sliding window length is obvious, with the difference between the F1 scores based on different window lengths being larger than 40%, regardless of the adopted machine-learning method. Performance generally improved with greater window lengths. However, the improvement became increasingly smaller and a cut-off window size emerged, after which the improvement was negligible. Based on the result, a window length between 2.5–3.5 s was proven to be the optimum value, given the tradeoff between recognition performance and speed, so this length is recommended for real-time applications with low latency requirements. As for applications that emphasize recognition performance, a window of six seconds is recommended.

#### 4.1.2. Motion Mode-Specific Analysis

**Figure 7:**
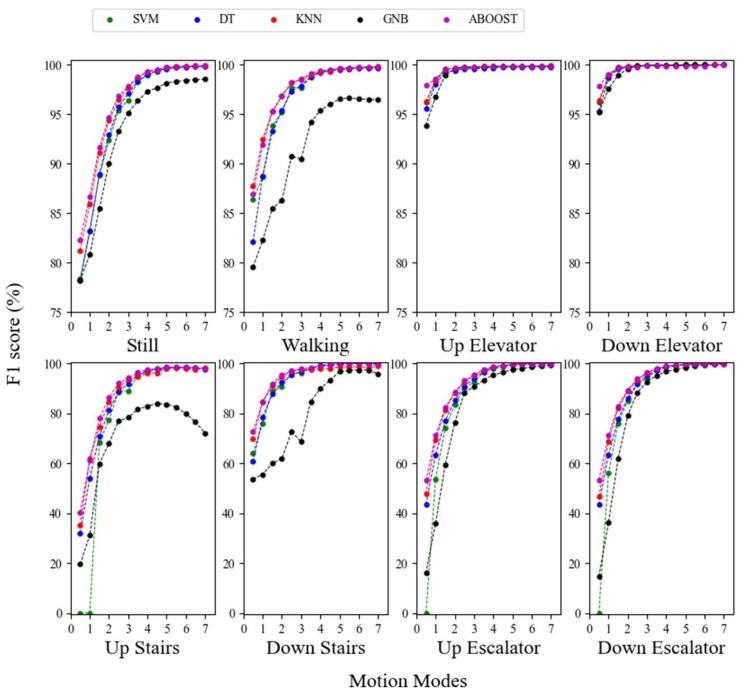
Relationship between F1 score and window length (horizontal axis) for different motion modes.

In addition to multiple motion mode recognition, specific motion mode recognition may also be required. Therefore, the impact of sliding window length on the specific motion mode was also examined. Figure 7 depicts the recognition results of a specific motion mode with different window lengths.

Figure 7 shows the impact of window length on every motion mode. The F1 score increased by expanding the window size by approximately 20% for still and walking detection, 5% for up and down elevator detection, 40% for up and down stairs detection, and notably, 60% for up and down escalator detection. The enhancement occurred with all of the methods.

In addition, the improvement caused by expanding the window size for specific motion mode detection became less distinct with the increase in window length, which is similar to the result obtained from the overall performance result analysis. However, different cut-off points for enhancement were observed for different motion modes. For instance, the main benefit of expanding the window size in up and down elevator detection occurred at a window size of 0.5 s to 1.5 s, with improvements of less than 0.5% after 1.5 s. However, the same was true after 3.5 s for still and working detection. Therefore, the suitable windows differ according to motion modes for users who are mainly concerned about specific motion mode recognition. Based on Figure 6, Table 5 summarizes the recommended window length for specific motion mode recognition.

As is shown in Figure 6 and Figure 7, a subtle reduction on performance requirement can generally allow us to evidently shorten the needed window length. This will be important for applications that require rapid detection, such as fall detection or indoor positioning [77]. Moreover, there are also other applications that further emphasize recognition performance such as an analysis of people’s movement in a whole day or counting the number of step in a day. Based on these different application needs, we listed the recommended window sizes that can guarantee different performance requirements (F1 score of 85% to 99%) in Table 5.

Table 5 also shows that taking elevators is an easily distinguishable motion mode. They can be recognized with a F1 score close to 94% with an interval of 0.5 s. To achieve similar performance, much larger windows are needed for the other motion modes, mainly because the high operating speed of elevators causes evident variation in air pressure, so that the classifiers can distinguish this mode from the others.

Conversely, the low operating speed of the escalator results in a much longer window to capture sufficient signal variation to achieve the same classification performance as with elevator classification. In elevator classification, any classifier with a 0.5-s window can operate with an F1 score of 94%. Nevertheless, the window length must be at least 2.5 s to obtain a similar performance for up and down escalator movement. Furthermore, the up and down elevator evaluations in Figure 6 had similar patterns, because their corresponding sensor signals have opposite signals and are approximately equal in magnitude. The same was true for the up and down escalator case.

To summarize, we explored the impact of sliding window length on specific motion mode recognition, and found that expanding the window evidently improved the recognition performance of each motion mode, regardless of the method adopted. The enhancement of the F1 score was over 50%. However, improvement by lengthening the sliding window becomes increasingly less with the expansion in window size, which renders blindly increasing window length for better performance unreasonable. Different enhancement cut-off window sizes exist for different motion modes. Based on the results, suitable window sizes are recommended according to the motion mode to be recognized and varying application needs.

### 4.2. Pose Classification Result

**Figure 8:**
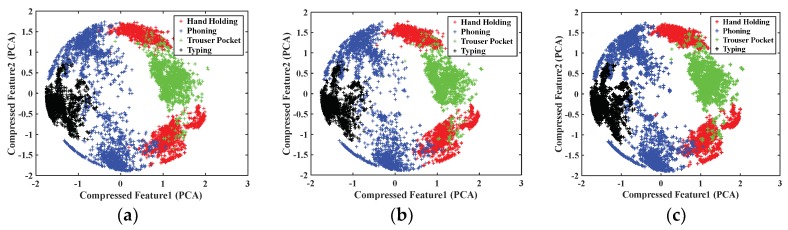
Distribution of compressed features of human poses with different window lengths: (**a**) 1 s; (**b**) 2 s; and (**c**) 3 s.

In this section, we analyze the effect of window length on human pose pattern classification and explore a suitable window length. First, we analyzed the effect of window length on the compressed pose pattern feature distribution. Figure 8 presents the results. The effect of window length on pose-specific features differed from that of motion mode. A change in boundaries among pose points was not evident as window length expanded. In contrast to motion mode classification, the effect of window length on human pose classification was limited.

#### 4.2.1. Global Evaluation

**Figure 9:**
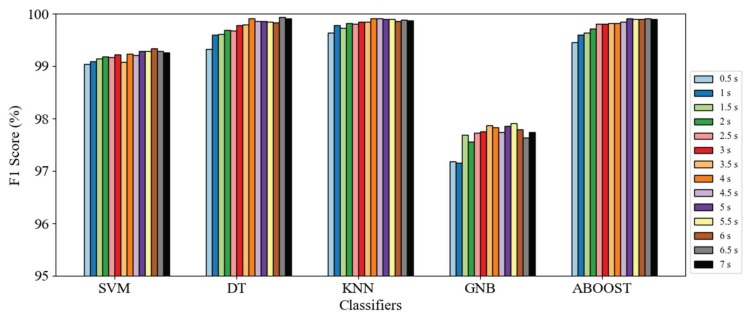
Average F1 score of pose classification using different machine-learning methods and window lengths.

In Figure 9, we compare the pose classification performance of each methodology with different window lengths. Each bar cluster represents different machine-learning methods, and bars in each cluster represent the average F1 score of poses based on window lengths of 0.5 s to 7 s. Among the classifiers, the performances of SVM, DT, KNN, and Adaboost were close to 99% based on a window of only 0.5 s. Although GNB performed the worst, it still achieved a score of 97%. The enhancement from expanding the window size was not evident (less than 0.8%), and a clear increasing trend in the F1 score was not apparent with the increase in window length.

#### 4.2.2. Pose-Specific Analysis

**Figure 10:**
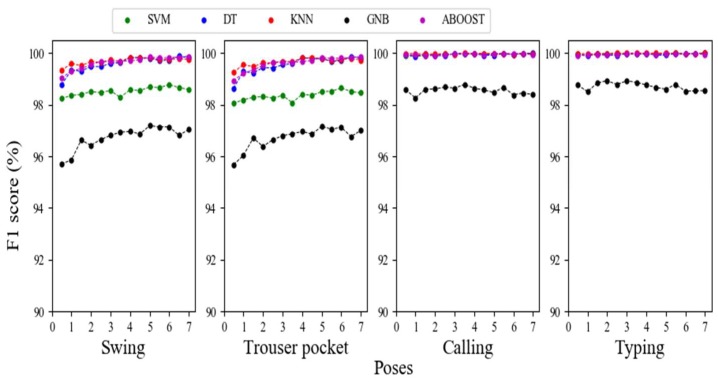
Relationship between F1 score and window length for pose classification.

Figure 10 depicts the details of classification performance for specific poses, with the F1 score for the poses using varying classification methods and window lengths. Initially, the classification F1 scores of ‘swing’ and ‘trouser pocket’ were similar, which also applied to calling and typing. We rationalize that when users are typing or calling, their pose patterns are unique, so they are easily distinguishable. Therefore, classifiers can recognize the pose patterns with an F1 score close to 100%. For ‘swing’, we found that people often held the smartphone in a similar manner as the smartphone being carried in the trouser pocket. In this case, although ‘swing’ and the trouser pocket can be distinguished from each other under dynamic motion mode, identifying the difference when the user was static was difficult. The confused samples between swing and trouser pocket when the user was static resulted in relatively poor performance for the swing and trouser pocket poses compared with typing and calling.

As depicted in Figure 10, an F1 score close to 99% was achieved based on a window of only 0.5 s for SVM, KNN, DT, and Adaboost in swing and trouser pocket classification. GNB performed the worst. However, GNB also received an F1 score higher than 95% with a 0.5-s window. For typing and calling classification, the results were even better. In these cases, every classifier performed impressively, and even the worst performance exceeded 98%. Therefore, for pose classification, a window of 0.5 s is sufficient, and using a longer window is unnecessary because the improvement is negligible and does not equal the sacrificed recognition speed. An F1 score beyond 95% was achieved based on a 0.5-s window with all of the classifiers.

In summary, in contrast to motion mode recognition, the influence of sliding window length on pose pattern recognition is not evident. Information extracted from gravity sensor data changes was sufficient to accurately classify these poses. Results show that a sliding window as short as 0.5 s can guarantee an F1 score higher than 95% for all of the pose patterns and machine-learning methods.

## 5. Discussion

Based on the findings, we propose some useful inferences, providing suggestions for future work. We also summarize the limitations of this work to improve our work in the future.

**Suggestions.** Even though it is easy to see from the standard workflow of HAR that sliding window length influences the HAR result directly, few researches have been done that reveal such impact in detail. Plenty of recent works [17,23,51,52] still keep selecting the window size intuitionally. However, determining the window length based on experience usually create bias in the results. For instance, the recognition performance of a similar motion mode group (walking, stationary, and going up and down stairs) with similar feature sets (time-domain and frequency-domain) was analyzed including the same method (random forest) [52,78]. However, the result presented in Qian et al. [78] exceeded the F1 score of 95%, whereas that in Yufei et al. [52] was less than 85%. Qian et al. attributed their good performance to a new strategy that they introduced in classification. However, they neglected that they used a sliding window length of 5 s, whereas Yufei et al. [52] used a short window of 1 s. Based on our study, the main reason for the improvement in Qian et al. [78] may have been caused by the much longer sliding window rather than the proposed strategy.

A detailed analysis in wearable sensor-based HAR has been presented by Banos et al. [67], but no studies have been conducted yet in smartphone sensor-based HAR. Smartphone-based HAR has different application contexts with wearable sensors [9,67,79]. Wearable sensors that attach to different parts of the human body permit measuring the motion experienced by each body limb and trunk, thus better capturing the human body dynamics. This guarantees the ability of wearable sensors in complex HAR such as sports activities [67,80], but the smartphone can only obtain the dynamic information of a certain part of the body, which makes it relatively weak. However, the smartphone has been extensively used in people’s daily life as the most popular device, and is more acceptable than wearable sensors for people to carry every day. Therefore, smartphone-based HAR is becoming increasingly popular in recognizing people’s daily activities [51,52,78,81]. As the results in [67] cannot apply to systems using smartphones, a comprehensive analysis is of concern in smartphone sensor-based HAR. Our research provides a comprehensive analysis in this field and makes up this gap.

Our study proves that the motion mode recognition result is influenced heavily by window size, which is independent of classification methods, and the influence on F1 score could be larger than 40%. For the users who largely require recognition performance, a longer window generally results in much better performance. However, blindly increasing the window size is also unreasonable because the improvement after a cut-off window length may be too small to be considered and not worth the sacrificed recognition speed. The improvement cut-off length is proved to be 6 s with an F1 score beyond 99%. As for users who largely focus on reducing recognition latency, they can shorten window sizes by reducing the required accuracy. In this case, a window between 2.5–3.5 s with an F1 score around 95% is recommended. In addition, the improvement cut-off points and the trade-off between performance and window length have been proven to differ according to motion modes. Therefore, window sizes fulfilling various accuracy requirements for specific motion mode recognition are listed in Table 5 for reference.

Our study of pose pattern recognition shows that the impact of window size on pose pattern recognition is limited, and gravity information proved effective in pose recognition even under various motion modes. Based on the variation in the gravity components on each axis of a smartphone local coordinate system, pose patterns can be classified accurately using a short window size of 0.5 s. This result provides a good reference for researchers who are interested in pose pattern recognition, especially in the field of indoor positioning.

In addition, results corroborate that Adaboost and KNN are more effective than other methods in motion mode and pose pattern recognition. GNB is not recommended based on the bad performance due to its simple principle and assumption that all of the input features are independent, so it cannot extract sufficient useful variation in the features to distinguish the activities. Based on this notion, Adaboost and KNN are recommended for use.

**Study generalization.** Regarding generalization, the tested recognition systems correspond with those that are widely used in related works. Furthermore, simplicity and comprehensiveness were key elements considered during our study, which enabled us to focus on the potential impact of segmentation on recognition. Thus, in this paper, the data directly captured through the sensors were used, thereby avoiding filtering or preprocessing. These procedures typically remove certain parts of raw signals, which potentially lead to a change in the signal space and limit the applicability of these results to other designs [67]. Moreover, time and frequency domain features were considered in our study to generalize our results, because these features were typically used in previous research [23,51,52]. The feature set extracted from different window sizes were kept constant to eliminate the potential bias from different feature sets on recognition to objectively present the impact of window size.

The motion modes and pose patterns considered in our research are common in people’s daily lives in indoor environments. We selected these motion modes and poses by observing human activities in a classical supermarket, which were also hot topics in previous studies [21,23,51,52].

As for the sensors used in our paper, accelerometers are widely used in HAR and have been proven to be effective in recognizing stationary and dynamic modes [64]. A barometer proved to be effective in recognizing vertical moving modes, such as the use of escalators and elevators. As for the elevator and escalator use cases, the subjects move in constant speed such that their acceleration is zero. However, the change in air pressure acts as an effective factor for classification. Therefore, the barometer is usually adopted in height-change motion mode recognition [64,65,66]. Thus, we used the accelerometer and barometer based on previous research suggestions to consider motion modes [64]. As for pose recognition, recognition based on gravity data has become increasingly popular because the variation in the gravity component on each axis of the smartphone under different poses is easily distinguishable [71]. The magnitude of gravity is constant, so that pose recognition will not be influenced by different motion modes. Therefore, the gravity sensor was used in our paper.

**Sampling rate.** Our results may also be influenced by the sampling rate. For this reason, we opted to define the window range in terms of time rather than sample amount. Maurer et al. [82] evaluated the effect of sampling rate on recognition accuracy, and found that evident gain did not exist for a sampling frequency over 20 Hz. Therefore, the results obtained could be, in principle, applied to other monitoring systems with sampling rates over 20 Hz.

**Performance metric.** In many studies, the recognition results of the system are normally measured in terms of accuracy or precision. Despite the extensive use of these metrics in many fields, they are biased in presenting the results, especially in the presence of imbalance issues in the experiment samples. Therefore, we adopted the F1 score in our work, which does not have this limitation [83]. Consequently, the results obtained could be generalized for each activity independent of the number of available instances for each target activity.

**Limitations.** Our work aimed to conduct a systematic evaluation of the impact of sliding window length on human motion mode and pose pattern recognition using smartphone motion sensors. However, we acknowledge that certain limitations are evident in our work.

1. Firstly, for motion mode recognition, neither a gyroscope nor a magnetometer were used. Although experiments have proven that the barometer and accelerometer are effective and sufficient, the current trends show that using additional sensors could help improve the recognition performance and system robustness. Therefore, an analysis using other smartphone sensors could be of interest and will be explored in future work.
2. Secondly, the dataset is relatively impoverished, because the data collection was taxing for researchers and subjects. Sufficient amounts of data could hardly be acquired over a short time period. In the future, we will recruit additional subjects so that our data will cover a wider range of ages, heights, and weights of the subjects, and so on. We also aim to establish a comprehensive human motion mode and pose pattern dataset for public use.
3. Finally, in this study, we mainly focused on revealing the impact of segmentation on HAR and manually tuning the window size. However, testing different window sizes before designing the system is time-consuming and inefficient. Advanced methods that could automatically tune the segmentation parameters based on the characteristics of the human activities to be distinguished would be considerably useful. Our future study will also focus on this aspect.

## 6. Conclusions

In human motion modes and pose pattern recognitions, windowing is a basic step used by the majority of scholars [64]. However, research largely relies on randomly selected values without careful analysis.

In this paper, we presented a comprehensive study that analyzed the influence of window length on human motion mode and pose pattern recognition. We evaluated the effect of window length on motion mode and pose pattern using five well-accepted classification methods. The results demonstrated that the window length affects motion mode recognition, but does not affect pose pattern recognition.

For motion mode classification, recognition performance generally improved by increasing the window length. However, the improvement became increasingly obscure, and a cut-off point was found to exist, after which improvement was negligible and not worth the sacrificed recognition speed. This result affirms that a window length between 2.5–3.5 s provides the best tradeoff between performance and latency. Adaboost performs the best in this window length range. Additionally, we proposed the recommended window lengths for use with varying motion mode classification requirements. In terms of pose classification, the effect of window length is limited, and the benefit of increasing the window length was less than 1% on the F1 score. All of the classification methods with 0.5-s windows achieved satisfactory results. In addition to the analysis on motion modes and pose cluster classification, the classification performance of specific motion modes and poses was analyzed. The suitable window length and technique can be determined through the use of experiments. The results provide a comprehensive understanding of the effect of window length on different classification methods, motion modes, and pose patterns, which subsequently determine the suitable window length and algorithm for motion modes and pose pattern recognition.

## Figures and Tables

**Table 1:** Subject information.

	Height (cm)	[163,170)	[170,175)	[175,180)
Weight (kg)				
[50,60)		Subject 1, 3		
[60,70)		Subject 8	Subject 2	Subject 4
[70,80)			Subject 9, 10	Subject 5, 6, 7

**Table 2:** Description of adopted smartphone sensors.

Sensors	Purpose	Data Stream	Description	Manufacturer	Measuring Range	Measuring Accuracy
Gravity sensor	Pose pattern classification	$G_{x}$	Gravity force along *x* axis	Qualcomm	39.226593 m/s^2^	0.00119 m/s^2^
		$G_{y}$	Gravity force along *y* axis			
		$G_{z}$	Gravity force along *z* axis			
Accelerometer	Motion mode classification	$A=\sqrt{A_{x}{}^{2}+A_{y}{}^{2}+A_{z}{}^{2}}$	$A_{*}$ is the specific force along * axis			
Barometer		P	Air pressure measurement	BOSCH	1100 hPa	0.00999 hPa

**Table 3:** Feature set.

No.#	FEATURE	DEFINITION
1	Mean	$mean(x)=\bar{x}=\frac{1}{N}\sum_{n=1}^{N}x[n]$
2	Absolute Mean	$mean(|x|)=\bar{\left|x\right|}$
3	Variance	$\mathrm{var}(x)=\sigma_{x}^{2}=\bar{{\left(x-\overset{¯¯}{x}\right)}^{2}}$
4	Standard deviation	$std(x)=\sigma^{x}=\sqrt{\mathrm{var}(x)}$
5	Mode	Values that appear most frequently in data set
6	Median	Middle value in a data set
7	Average Absolute Difference	$mean(|x^{-}|)=\bar{\left|x-\bar{x}\right|}$
8	75th Percentile	Value separating 25% higher data from 75% lower data in a data set.
9	Interquartile range	Difference between 75th and 25th percentile
10	Gradient (only for air pressure data)	The coefficient of first-order linear fitting
11	Coefficients of FFT (Fast Fourier Transform)	Energy of each frequency component

**Table 4:** Confusion matrix in classifying class A.

		Predicted Class	
		A	B
**Actual Class**	**A**	TP	FN
	**B**	FP	TN

**Table 5:** Recommended window size for specific motion mode classification.

Motion Mode	Recommended Window Size			
	F1 Score			
	85%	90%	95%	99%
Stationary	1.5 s	2 s	3 s	4.5 s
Walking	1 s	1.5 s	3 s	4 s
Up elevator	0.5 s	0.5 s	0.5 s	1.5 s
Down elevator	0.5 s	0.5 s	0.5 s	1.5 s
Up stairs	2 s	3 s	3.5 s	5 s
Down stairs	1.5 s	2 s	2.5 s	4 s
Up escalator	2 s	2.5 s	3.5 s	4.5 s
Down escalator	2 s	2.5 s	3 s	4.5 s
